# Alteration of Prion Strain Emergence by Nonhost Factors

**DOI:** 10.1128/mSphere.00630-19

**Published:** 2019-10-09

**Authors:** Sara A. M. Holec, Qi Yuan, Jason C. Bartz

**Affiliations:** a Department of Medical Microbiology and Immunology, School of Medicine, Creighton University, Omaha, Nebraska, USA; Colorado State University

**Keywords:** prion, strain, strain emergence

## Abstract

Prions can persist in the environment for extended periods of time after adsorption to surfaces, including soils, feeding troughs, or fences. Prion strain- and soil-specific differences in prion adsorption, infectivity, and response to inactivation may be involved in strain maintenance or emergence of new strains in a population. Extensive proteinase K (PK) digestion of Hyper (HY) and Drowsy (DY) PrP^Sc^ resulted in a greater reduction in the level of DY PrP^Sc^ than of HY PrP^Sc^. Use of the PK-digested material in protein misfolding cyclic amplification strain interference (PMCAsi) resulted in earlier emergence of HY PrP^Sc^ than of undigested controls. This result established that strain-specific alteration of the starting ratios of conversion-competent HY and DY PrP^Sc^ can alter strain emergence. We next investigated whether environmentally relevant factors such as surface binding and weathering could alter strain emergence. Adsorption of HY and DY PrP^Sc^ to silty clay loam (SCL), both separately and combined, resulted in DY interfering with the emergence of HY in PMCAsi in a manner similar to that seen with unbound controls. Similarly, repeated cycles of wetting and drying of SCL-bound HY and DY PrP^Sc^ did not alter the emergence of HY PrP^Sc^ compared to untreated controls. Importantly, these data indicate that prion strain interference can occur when prions are bound to surfaces. Interestingly, we found that drying of adsorbed brain homogenate on SCL could restore its ability to interfere with the emergence of HY, suggesting a novel strain interference mechanism. Overall, these data provide evidence that the emergence of a strain from a mixture can be influenced by nonhost factors.

**IMPORTANCE** The prion strain, surface type, and matrix containing PrP^Sc^ can influence PrP^Sc^ surface adsorption. The cumulative effect of these factors can result in strain- and soil-specific differences in prion bioavailability. Environmental weathering processes can result in decreases in PrP^Sc^ conversion efficiency and infectivity. Little is known about how incomplete inactivation of surface-bound PrP^Sc^ affects transmission and prion strain emergence. Here, we show that strain interference occurs with soil-bound prions and that altering the ratios of prion strains by strain-specific inactivation can affect strain emergence. Additionally, we identify a novel mechanism of inhibition of prion conversion by environmental treatment-induced changes at the soil-protein interface altering strain emergence. These novel findings suggest that environmental factors can influence strain emergence of surface-bound prions.

## Abstract (precis)

The prion strain, surface type, and matrix containing PrP^Sc^ can influence PrP^Sc^ surface adsorption. The cumulative effect of these factors can result in strain- and soil-specific differences in prion bioavailability. Environmental weathering processes can result in decreases in PrP^Sc^ conversion efficiency and infectivity. Little is known about how incomplete inactivation of surface-bound PrP^Sc^ affects transmission and prion strain emergence. Here, we show that strain interference occurs with soil-bound prions and that altering the ratios of prion strains by strain-specific inactivation can affect strain emergence. Additionally, we identify a novel mechanism of inhibition of prion conversion by environmental treatment-induced changes at the soil-protein interface altering strain emergence. These novel findings suggest that environmental factors can influence strain emergence of surface-bound prions.

## INTRODUCTION

Prion diseases are emerging zoonotic transmissible neurodegenerative disorders of animals, including humans. The causative agent of prion disease is PrP^Sc^, a misfolded isomer of the normal cellular prion protein, PrP^C^ (1–5). Prion diseases can have a familial, sporadic, or infectious etiology and, with no effective treatments, are inevitably fatal. Prion conversion occurs when PrP^Sc^ binds to PrP^C^, and, through an unknown mechanism, PrP^Sc^ directs the conversion of PrP^C^ to PrP^Sc^ (6, 7). This process is shared among several protein misfolding diseases, including Parkinson’s and Alzheimer’s diseases (8, 9). Prion strains can differ in neuropathology, incubation period, host range, and pathogenicity and are hypothesized to be encoded by strain-specific conformations of PrP^Sc^ (10–17).

Prions adsorb to surfaces and remain infectious. Prions can contaminate surgical instruments and remain infectious following standard sterilization procedures, enabling iatrogenic transmission in medical settings (18, 19). In prion diseases of sheep and cervids, prions can be shed into the environment via a variety of biological matrices and can bind to surfaces, including soils, plants, feeding troughs, and fences (20–27). The adsorption of PrP^Sc^ to a surface is influenced by numerous factors, including the prion strain, the species of origin, the biological matrix that contains PrP^Sc^, and the surface type, with no predictable behavior for any singular criterion (20, 22, 28, 29). Prions in the environment can remain infectious for extended periods of time as indicated by transmission of chronic wasting disease (CWD) and scrapie to cervids and sheep, respectively, in pastures unoccupied by prion-infected animals for many years (30, 31).

Prion exposure to external treatments can alter PrP^Sc^ properties. Standard sterilization procedures, such as autoclaving, can partially inactivate PrP^Sc^ adsorbed to medical instruments (32–34). Exposure of soil-bound PrP^Sc^ to repeated cycles of wetting and drying can reduce PrP^Sc^ abundance and protein misfolding cyclic amplification (PMCA) conversion efficiency and prion infectivity (35). Importantly, the effect of weathering on prion infectivity can vary with prion strain as well as soil type (35). Prions bound to soil and surgical instruments may undergo similar structural changes that facilitate disease transmission. For example, dehydration of PrP^Sc^ on soil and stainless-steel surgical tools was previously shown to render the protein less sensitive to weathering and decontamination processes, respectively (36–38). Little is known about how strain-specific incomplete inactivation of surface-bound PrP^Sc^ affects strain emergence.

Many factors can influence the emergence of a dominant strain from a mixture. When a host is infected with multiple prion strains, interference can occur where a slowly converting strain delays or blocks the emergence of a quickly converting strain (39–42). The relative times of onset of conversion of two strains in the same cell govern the outcome of strain interference (41). Mechanistically, this is due to prion strains competing for a limiting cellular resource, PrP^C^ (43). The emergence of a strain from a mixture can be influenced by the ratio of the prion strains that initially infected the host (41, 44, 45). While it is known that strain selection can occur within a prion-infected host, it is unknown if environmental factors would favor the survival and transmission of a subset of strains that are introduced into the environment. Here, we investigate the effect of simulated weathering and degradation conditions on prion stain emergence.

## RESULTS

### Increased sensitivity of DY PrP^Sc^ to degradation compared to HY PrP^Sc^.

**FIG 1:**
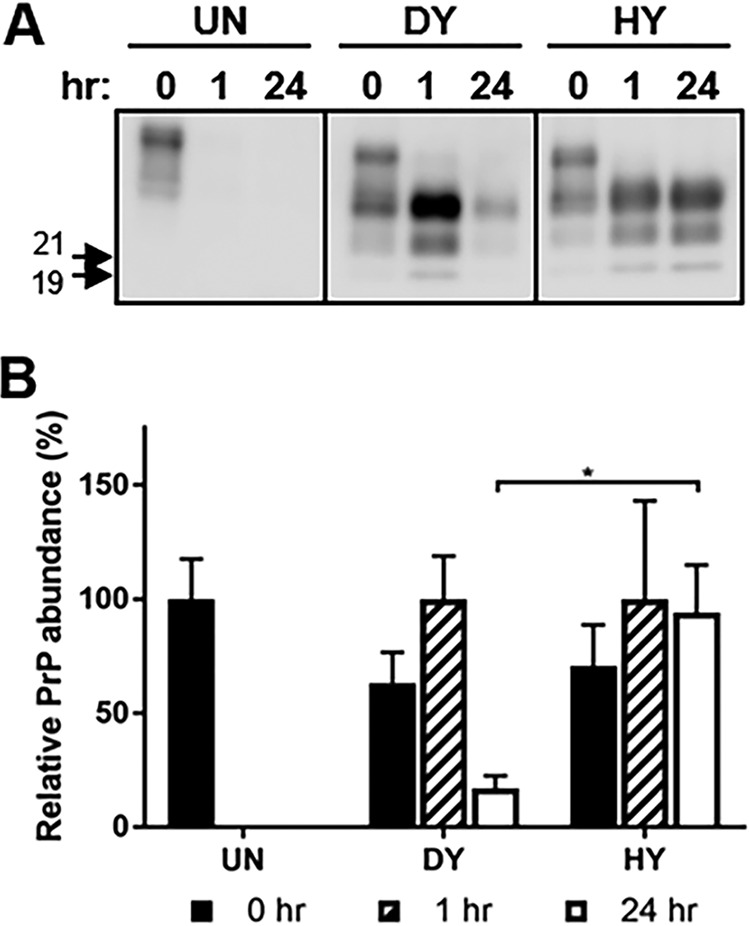
Strain-specific sensitivity to proteolytic digestion. Western blotting (A) and quantification (B) of PrP from mock-infected (UN), DY-infected, or HY-infected brain homogenate treated without (0) or with (1 or 24 h) proteinase K (PK). The abundance of PK-digested DY PrP^Sc^ was significantly (*P* < 0.05) reduced compared to that of PK-digested HY PrP^Sc^ (*n* = 3). Migration of 19-kDa and 21-kDa molecular weight marker is indicated on the left of the Western blot. Statistical analysis: Student's *t* test; *, *P* < 0.05; *n* = 3. This experiment was repeated a minimum of 3 times with similar results.

Following digestion of brain homogenate with proteinase K (PK), we failed to detect PrP^C^ in the uninfected brain homogenate (Fig. 1A). Digestion of Hyper (HY)- or Drowsy (DY)-infected brain homogenates with PK resulted in N-terminal truncation of PrP^Sc^ with the characteristic strain-specific migration of the unglycosylated PrP^Sc^ polypeptide at 21 or 19 kDa, respectively (46) (Fig. 1A). A 24-h PK digestion of DY-infected brain homogenates (*n* = 3) resulted in an 80% reduction in PrP^Sc^ abundance, and digestion of HY-infected brain homogenates (*n* = 3) resulted in a 5% reduction in PrP^Sc^ abundance, compared to 1-h PK digests of DY and HY, respectively (Fig. 1B). DY PrP^Sc^ was significantly (*P* < 0.05) more sensitive to PK digestion than HY PrP^Sc^ (Fig. 1B).

### Preferential removal of DY PrP^Sc^ enhances the emergence of HY PrP^Sc^.

**FIG 2:**
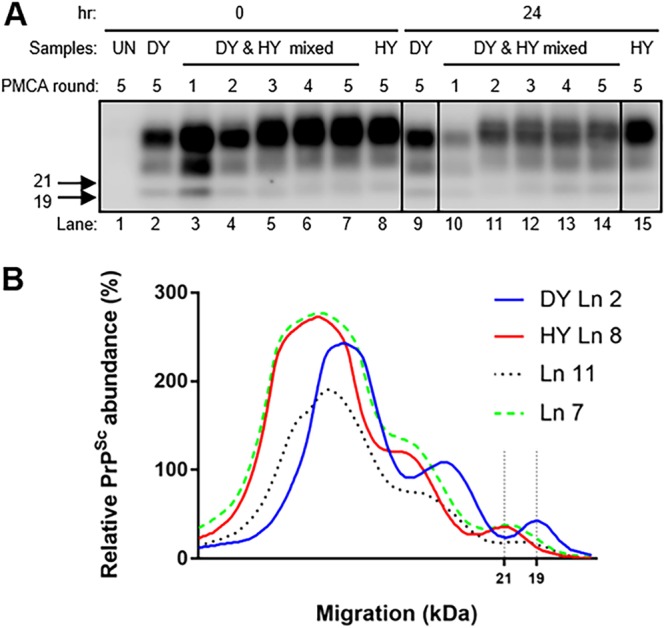
Selective degradation of DY PrP^Sc^ enhances the emergence of HY PrP^Sc^ from a mixture. Western blotting (A) and migration analysis (B) of PrP^Sc^ from PMCAsi reactions. Negative-control brain homogenate reactions did not amplify PrP^Sc^ (lane [Ln] 1). Positive-control PMCA reaction mixtures seeded with either HY-infected (lane 8) or DY-infected (lane 2) brain homogenate maintained the 21-kDa (lane 15) or 19-kDa (lane 9) strain-specific unglycosylated PrP^Sc^ migration pattern, respectively, following 5 rounds of amplification. Positive-control PMCAsi reaction mixtures seeded with both HY and DY (lanes 3 to 7) in the absence of PK digestion resulted in HY PrP^Sc^ emerging by round 5. Experimental PMCAsi reaction mixtures seeded with both HY and DY (lanes 10 to 14) that were digested with PK for 24 h resulted in HY PrP^Sc^ emerging in round 2. Migration of 19-kDa and 21-kDa molecular weight marker is indicated on the left of the Western blot. This experiment was repeated a minimum of 3 times with similar results.

To determine if altering the ratio of HY and DY by selective degradation could alter strain emergence, PK-digested or mock-digested HY-infected and DY-infected brain homogenates were used as seeds for PMCA strain interference (PMCAsi). Positive non-PK-digested control PMCA reaction mixtures seeded with either 0.05 μg eq of HY-infected or 500 μg eq DY-infected brain homogenate resulted in amplification of PrP^Sc^ that maintained the strain-specific migration pattern (Fig. 2). Unseeded negative-control PMCA reaction mixtures did not amplify PrP^Sc^ (Fig. 2A). To test strain interference *in vitro*, 0.05 μg eq of HY-infected and 500 μg eq of DY-infected brain homogenates were mixed together as the seed for PMCAsi. This ratio of HY-infected and DY-infected brain homogenate was used for all described PMCAsi reactions and was chosen based on past PMCAsi experiments (43). In the strain interference positive-control group, HY PrP^Sc^ emerged after 4 rounds of PMCA (Fig. 2). In the PK-digested experimental group (Fig. 2), HY PrP^Sc^ emerged after 2 rounds of PMCAsi (Fig. 2). Overall, alteration of the effective ratio of HY to DY PrP^Sc^ by PK digestion results in the earlier emergence of HY PrP^Sc^.

### The PMCA conversion activity of PK-digested PrP^Sc^ is not altered.

**FIG 3:**
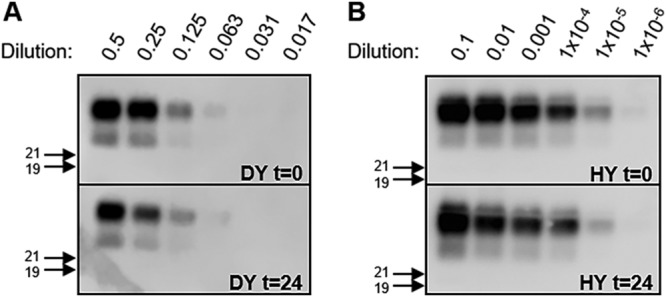
PMCA conversion activity per unit of PrP^Sc^ is unchanged by PK digestion. Results of Western blotting of PrP^Sc^ from DY (A) or HY (B) PMCA reaction mixtures seeded with either untreated DY or HY (t = 0) or the same amount of PrP^Sc^ remaining after 24 h of PK digestion (t = 24) are shown. Migration of 19-kDa and 21-kDa molecular weight marker is indicated on the left of the Western blot. This experiment was repeated a minimum of 3 times with similar results.

PK-digested HY PrP^Sc^ may have an increased level of PMCA conversion activity per unit PrP^Sc^ compared to mock-digested PrP^Sc^ that may contribute to the earlier emergence of HY PrP^Sc^ in PMCAsi. To test this possibility, determinations of the PMCA conversion coefficient (PMCA-CC) of equal amounts of PK-digested and undigested PrP^Sc^, as determined by Western blotting, were performed. Following one round of PMCA, the PK-digested PrP^Sc^ and mock-digested DY PrP^Sc^ both amplified to a dilution factor of 0.0625 (Fig. 3A) and the mock-digested PrP^Sc^ and PK-digested HY PrP^Sc^ both amplified to a dilution factor of 1 × 10^−5^ (Fig. 3B). Overall, we found that the PMCA conversion activity of the PrP^Sc^ that remained after PK digestion was similar to that of the undigested PrP^Sc^.

### Prions bound to soil participate in strain interference.

**FIG 4:**
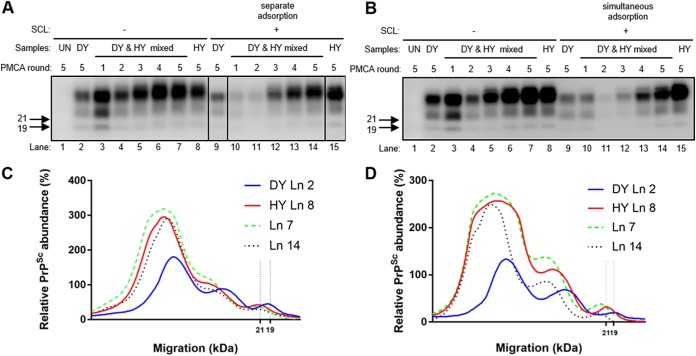
Binding of PrP^Sc^ to SCL does not alter strain emergence from a mixture. Results of Western blotting (A and B) and migration analysis (C and D) of PrP^Sc^ from PMCAsi reaction mixtures seeded with HY and DY bound separately to SCL (A and C) or bound to SCL as a mixture (B and D) are shown. Negative-control brain PMCA reactions did not amplify PrP^Sc^ (A and B, lanes 1). Positive-control PMCA reaction mixtures seeded with either HY-infected (lane 8) or DY-infected (lane 2) brain homogenate maintained the 21-kDa (lane 15) or 19-kDa (lane 9) strain-specific unglycosylated PrP^Sc^ migration pattern, respectively, after 5 rounds of amplification. Positive-control PMCAsi reaction mixtures seeded with both unbound HY and DY resulted in the emergence of HY PrP^Sc^ at round 5 (lanes 3 to 7). Experimental PMCAsi reaction mixtures seeded with both SCL-HY and SCL-DY bound separately (A, lanes 10 to 14) or together (B, lanes 10 to 14) resulted in HY PrP^Sc^ emerging by round 5. Migration of 19-kDa and 21-kDa molecular weight marker is indicated on the left of the Western blot. This experiment was repeated a minimum of 3 times with similar results.

Prions can bind to a variety of soil types, and adsorption to silty clay loam (SCL) decreases PMCA-CC of HY and DY PrP^Sc^ (22, 47). To investigate the effects of this environmentally relevant condition on strain emergence, HY and DY was adsorbed to SCL (SCL-HY and SCL-DY, respectively) prior to five rounds of PMCAsi. After five rounds of PMCAsi, PrP^Sc^ was not observed in the negative-control samples, and HY and DY positive-control PMCA reactions maintained the strain-specific migration of 21- and 19-kDa PrP^Sc^, respectively (Fig. 4, lanes 1, 2, 8, 9, and 15). When DY and HY PrP^Sc^ were bound to SCL, either separately (Fig. 4A) or together (Fig. 4B), DY PrP^Sc^ was able to interfere with the emergence of HY PrP^Sc^ similarly to unbound control PMCAsi reactions (Fig. 4). Overall, SCL-bound prions were able to participate in strain interference and binding of HY and DY PrP^Sc^ to silty clay loam did not alter strain emergence under the conditions tested.

### Repeated cycles of dehydration and rehydration of unbound prions do not alter prion strain emergence.

**FIG 5:**
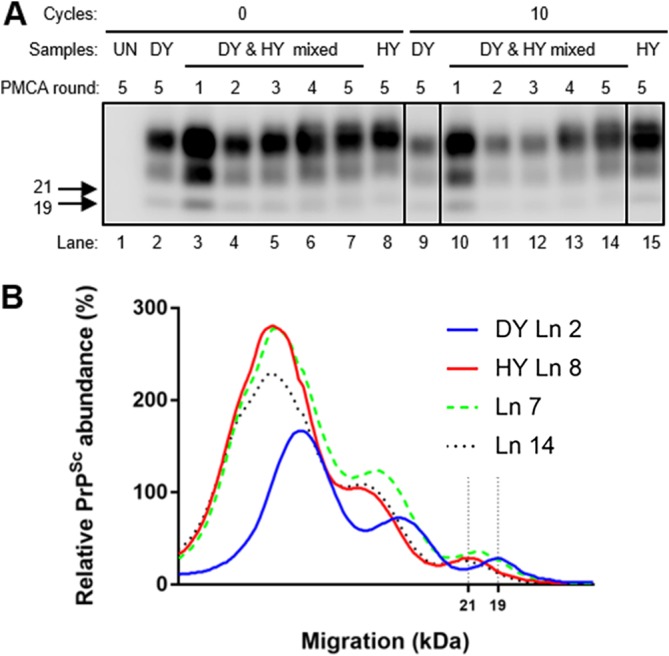
Repeated cycles of dehydration and hydration does not alter strain emergence. Western blotting (A) and migration analysis (B) of PrP^Sc^ from PMCAsi reactions. Negative-control PMCA reactions did not amplify PrP^Sc^ (lane 1). Positive-control PMCA reaction mixtures seeded with either HY-infected (lane 8) or DY-infected (lane 2) brain homogenate amplified PrP^Sc^ that maintained the 21-kDa (lane 15) or 19-kDa (lane 9) strain-specific unglycosylated PrP^Sc^ migration pattern, respectively, after 5 rounds of amplification. Positive-control zero wetting and drying cycle PMCAsi reaction mixtures seeded with both HY and DY (lanes 3 to 7) resulted in the emergence HY PrP^Sc^ by round 5. Experimental PMCAsi reaction mixtures seeded with both HY and DY after 10 serial rounds of wetting and drying treatment (lanes 10 to 14) resulted in the emergence of HY PrP^Sc^ by round 5. Migration of 19-kDa and 21-kDa molecular weight marker is indicated on the left of the Western blot. This experiment was repeated a minimum of 3 times with similar results.

In the environment, prions encounter various weathering processes, including repeated cycles of dehydration and rehydration (referred to as “treated” in the following experiment descriptions). To investigate whether a natural weathering process can affect strain emergence, 0.05 μg eq of unbound HY and 500 μg eq of unbound DY PrP^Sc^ were mixed prior to five rounds of PMCAsi. PrP^Sc^ was not detected in negative-control reactions after five rounds of PMCA, and HY-seeded and DY-seeded positive-control PMCA reaction mixtures maintained the strain-specific migration of 21- and 19-kDa PrP^Sc^, respectively (Fig. 5A). In control untreated samples, HY emerged in round 4 of PMCAsi (Fig. 5B). When unbound HY and DY were exposed to 10 repeated cycles of wetting and drying treatment (t = 10) prior to PMCAsi, HY emerged in round 4 of PMCAsi similarly to untreated control PMCAsi reactions (Fig. 5B). Overall, 10 cycles of wetting and drying treatment did not alter the emergence of unbound HY in PMCAsi.

### Repeated cycles of wetting and drying of brain homogenate to silty clay loam alter prion strain emergence.

**FIG 6:**
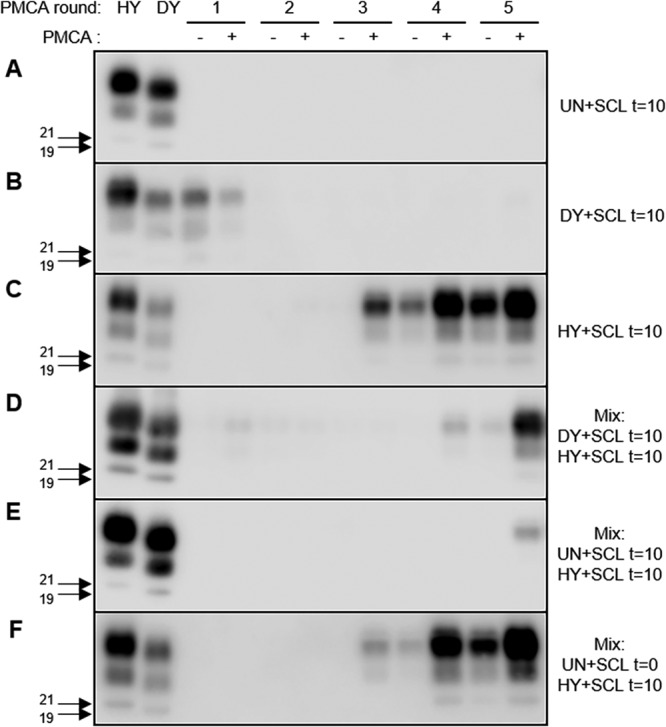
Drying of brain homogenate to soil restores strain interference properties (see also Fig. S1). Representative results of Western blotting of PrP^Sc^ amplificiation before (−) or after (+) PMCA analysis of UN (A), DY (B), HY (C), mixed DY and HY (D), and mixed UN and HY after zero (t = 0) (F) or 10 (t = 10) (A, B, C, D, and E) cycles of wetting and drying are shown. Migration of 19-kDa and 21-kDa molecular weight marker is indicated on the left of the Western blot. This experiment was repeated a minimum of 3 times with similar results.

Adsorbing prions to soil before dehydration and rehydration cycles protects PrP^Sc^ from degradation but results in a significant decrease in PMCA conversion efficiency (35). To examine if this decrease in conversion efficiency would alter strain emergence *in vitro*, 10 serial rounds of wetting and drying were performed on SCL-uninfected, SCL-HY-infected, or SCL-DY-infected brain homogenate. Negative-control PMCA reaction mixtures containing uninfected brain homogenate bound to SCL did not amplify PrP^Sc^ (Fig. 6A). PMCA reaction mixtures containing SCL-DY treated with 10 serial rounds of wetting and drying did not result in detection of PrP^Sc^ after five rounds (Fig. 6B). In samples containing treated SCL-HY, detectable conversion occurred as early as round 3 of PMCA (Fig. 6C). PMCAsi reaction mixtures containing treated SCL-DY and SCL-HY resulted in detection of HY PrP^Sc^ at round 4 (Fig. 6D), suggesting that in the absence of DY PrP^Sc^ conversion (Fig. 6B), HY PrP^Sc^ emergence was delayed (Fig. 6D) compared to the results seen with PMCA reaction mixtures containing treated SCL-HY alone (Fig. 6C). To test whether this phenomenon were specific to dehydrated SCL-DY, PMCAsi reaction mixtures were seeded with treated SCL-uninfected brain homogenate (Fig. 6A) or treated SCL-HY (Fig. 6C) and resulted in HY PrP^Sc^ emergence at round 5 (Fig. 6E). This suggested that the interference effect was independent of DY PrP^Sc^. To test if this was due to the hydration state of the brain homogenate, PMCAsi reaction mixtures were seeded with SCL-uninfected brain homogenate without wetting and drying treatment and treated SCL-HY (Fig. 6C). This resulted in the emergence of HY PrP^Sc^ in round 3 (Fig. 6F) similarly to PMCA reaction mixtures containing only treated SCL-HY (Fig. 6C). Overall, these data suggest that dried, surface-adsorbed brain homogenate can delay the emergence of HY PrP^Sc^.

10.1128/mSphere.00630-19.1 FIG S1 Drying of brain homogenate to soil restores strain interference properties. Quantification of PrP^Sc^ abundance was performed before (−) or after (+) PMCA analysis of UN (A), DY (B), HY (C), mixed DY and HY (D), and mixed UN and HY after zero (t = 0) (F) or 10 (t = 10) (A, B, C, D, and E) wetting and drying cycles. Results of analysis of PrP^Sc^ abundance after PMCA analysis of UN, DY, HY, mixed DY and HY, and mixed UN and HY after zero (t = 0) or 10 (t = 10) cycles of wetting and dryings are shown. Statistical analysis: Student’s *t* test; *, *P* < 0.05; *n* = 3. This experiment was repeated a minimum of 3 times with similar results. Download FIG S1, TIF file, 0.4 MB. Copyright © 2019 Holec et al. 2019 Holec et al. This content is distributed under the terms of the Creative Commons Attribution 4.0 International license.

### SCL-bound prions retain infectivity after wetting and drying treatment.

To investigate the effect of repeated cycles of wetting and drying on unbound and SCL-bound HY or DY infectivity, the incubation period and attack rate of groups of 5 hamsters per inoculum were determined. Negative-control hamsters inoculated with uninfected hamster brain homogenate did not develop clinical signs of prion disease at 240 days postinfection (p.i.), when the experiment was terminated (Table 1). Brain material from these animals did not contain detectable PrP^Sc^ by Western blotting (see Fig. S2 in the supplemental material). All of the hamsters inoculated with untreated or treated unbound HY-infected brain homogenate developed clinical signs of hyperexcitability and ataxia at 62 ± 3 or 73 ± 3 days p.i., respectively. All of the animals inoculated with untreated or treated SCL-bound HY-infected brain homogenate developed clinical signs of hyperexcitability and ataxia at 81 ± 3 or 96 ± 3 days p.i., respectively (Table 1). Brain material from all hamsters that developed clinical signs of hyperexcitability and ataxia contained an unglycosylated PrP^Sc^ polypeptide that migrates at 21 kDa, consistent with HY infection (Fig. S2). In the HY-infected animals, 10 cycles of wetting and drying, binding to SCL, and the combination of SCL binding and treatment resulted in a significant (*P* < 0.0001) increase in the incubation period compared to untreated controls (Table 1).

**TABLE 1:** Incubation period and attack rate of unbound and SCL-bound prions^*a*^

Inoculum	A/I^*b*^	Incubation period (days)^*c*^
UN	0/5	>240
HY at *t* = 0	5/5	62 ± 3
HY at *t* = 10	5/5	73 ± 3
HYSCL at *t* = 0	5/5	81 ± 3
HYSCL at *t* = 10	5/5	96 ± 3
DY at *t* = 0	5/5	176 ± 3
DY at *t* = 10	5/5	185 ± 3
DYSCL at *t* = 0	5/5	217 ± 4
DYSCL at *t* = 10	5/5	221 ± 3

a DYSCL, DY PrP^Sc^ adsorbed to silty clay loam; HYSCL, HY PrP^Sc^ adsorbed to silty clay loam; UN, mock infection.

b Number affected/number inoculated.

c Time from inoculation to onset of clinical signs (mean incubation period ± standard errors of the means [SEM]).

10.1128/mSphere.00630-19.2 FIG S2 Western blotting confirmation of clinical diagnosis of prion disease. Western blotting was performed on PK-digested brain homogenate from representative animals from each experimental group reported in Table 1. Brain material from animals inoculated with HY- or DY-infected samples contained the characteristic 21-kDa or 19-kDa migration of the unglycosylated PrP^Sc^ polypeptide of HY or DY, respectively. Brain homogenate from mock-infected (UN) animals did not contain detectable PrP^Sc^. Migration of 19- and 21-kDa-molecular-weight markers is indicated on the left of the Western blot. Download FIG S2, TIF file, 0.1 MB. Copyright © 2019 Holec et al. 2019 Holec et al. This content is distributed under the terms of the Creative Commons Attribution 4.0 International license.

All of the hamsters inoculated with untreated or treated bound DY-infected brain homogenate developed clinical signs of progressive lethargy at 176 ± 3 or 185 ± 3 days p.i., respectively, and untreated or treated SCL-DY-infected brain homogenate developed clinical signs of lethargy at 217 ± 3 or 221 ± 3 days p.i., respectively (Table 1). Brain material from all hamsters that developed clinical signs of lethargy contained an unglycosylated PrP^Sc^ polypeptide that migrates at 19 kDa, consistent with DY infection (Fig. S2). In the DY-infected animals, binding to SCL resulted in a significant (*P* < 0.0001) extension in the length of the incubation period; however, 10 cycles of wetting and drying did not significantly (*P* = 0.1986 and *P* = 0.7654, respectively) extend the incubation period in both the unbound and SCL-bound groups compared to untreated controls (Table 1).

## DISCUSSION

Prion strain interference can occur when PrP^Sc^ is bound to a surface. Environmental transmission of prions in cervids and sheep can involve PrP^Sc^ bound to soil, and iatrogenic prion diseases of humans can be transmitted by PrP^Sc^ bound to stainless steel surgical instruments (48). The effect of prions binding to surfaces on strain selection is unknown. Here, we show that when HY PrP^Sc^ and DY PrP^Sc^ are bound to SCL, either separately or as a mixture, SCL-DY PrP^Sc^ can interfere with the emergence of SCL-HY PrP^Sc^ similarly to unbound control PMCAsi reactions (Fig. 3). These data suggest that soil-bound PrP^Sc^ can compete for PrP^C^, which is thought to be the limiting factor in strain interference (43, 49). We hypothesize that the PrP^C^ binding site on PrP^Sc^ is different from the site at which PrP^Sc^ binds to the soil surface, allowing adequate conversion activity during the initial round of PMCAsi, when PrP^Sc^ exists largely in the adsorbed state (50, 51). This observation suggests that strain interference between different strains of soil-bound prions in natural settings can influence the emergence of a strain from a mixture.

Altering the ratio of prion strains in a mixture by strain-specific selective degradation can alter prion strain emergence. The increased susceptibility of DY PrP^Sc^ to enzymatic degradation compared to HY PrP^Sc^ resulted in earlier emergence of HY PrP^Sc^ *in vitro* (Fig. 2). This result suggests that in the environment, PrP^Sc^ from strains that can survive environmental weathering conditions may be more likely to be transmitted to a new host and therefore be favored in a population. However, it is possible that the subpopulation of PrP^Sc^ that survives has an increased titer per unit PrP^Sc^ and that this may explain the observed results. To investigate this possibility, we determined the PMCA conversion activity of PrP^Sc^ after digestion and found similar levels of PMCA conversion efficiency of undigested and digested samples when normalized for PrP^Sc^ abundance. This indicates that the subpopulation of PrP^Sc^ that survives digestion has conversion activity similar to that of the untreated controls. This is consistent with a previous report indicating that PK digestion does not alter strain properties (10). Therefore, we conclude that the enhanced emergence of HY following PK digestion is explained solely by the relatively larger decrease in the amount of DY PrP^Sc^, compared to HY PrP^Sc^, seeding the initial PMCA reaction mixture. This finding that partial inactivation of prions may allow the emergence of a more highly pathogenic strain may also provide mechanistic insight into the emergence of thermostable strains following incomplete inactivation of prions during rendering which may have contributed to the emergence of bovine spongiform encephalopathy (52–58).

Repeated cycles of dehydration and rehydration can affect prion strain emergence. Environmental weathering processes such as wetting and drying or freezing and thawing can decrease PrP^Sc^ conversion efficiency and enhance PrP^Sc^ degradation (35, 36); however, it is unknown if these changes can alter the emergence of a strain from a mixture. Here, we show that the emergence of HY from a mixture exposed to 10 repeated cycles of wetting and drying was unaltered compared to untreated controls (Fig. 4). Therefore, the relative decrease in PrP^Sc^ conversion efficiency between HY and DY was insufficient to affect the emergence of HY. Interestingly, we found that 10 serial rounds of wetting and drying of SCL-DY did not alter the abundance of DY PrP^Sc^ but resulted in an extinction of PMCA conversion activity under the conditions tested (PMCA conversion-incompetent SCL-DY) (Fig. 6). This observation of preservation of a PK-resistant population of SCL-bound PrP^Sc^ that had reduced conversion activity with repeated cycles of wetting and drying or freezing and thawing was previously observed (35, 36). On the basis of this observation, we reasoned that PMCA conversion-incompetent SCL-DY would be unable to inhibit HY conversion, thereby allowing HY to emerge more rapidly when present in a mixture. This would also be consistent with our findings showing selective digestion of DY PrP^Sc^ allowing the more rapid emergence of HY PrP^Sc^ (Fig. 1 and 2). Unexpectedly, we found that the presence of PMCA conversion-incompetent SCL-DY was able to interfere with the emergence of HY PrP^Sc^ similarly to positive-control PMCA replication-competent populations of DY PrP^Sc^ (Fig. 6). To investigate the mechanism of this finding, we found that 10 serial rounds of wetting and drying of SCL-uninfected brain homogenate had the same interference effect as that seen with the conversion-incompetent SCL-DY. While prions are not present in uninfected brain homogenate, the dried SCL-adsorbed homogenate was able to interfere with HY emergence, suggesting a prion-independent effect. Importantly, SCL-uninfected brain homogenate without serial rounds of wetting and drying and SCL alone did not have this effect (Fig. 6). These data suggest that dehydration of brain homogenate at the soil surface may allow further protein adsorption, leading to the observed delay in HY PrP^Sc^ emergence. Soil in areas with high animal density such as salt licks and feeding troughs is frequently exposed to prion shedding. The infectivity of soil in these areas may be higher due to additional surface binding of protein after cycles of drying and wetting. Overall, incomplete degradation of PrP^Sc^ and the physical state of a protein bound to a surface can influence the effective ratios of strains in the environment and affect the emergence of a strain from a mixture.

Prions bound to SCL and treated with 10 serial rounds of wetting and drying remain infectious. Changes in the infectivity of bound and unbound treated HY PrP^Sc^ were consistent with a previous report (35). PMCA conversion-incompetent SCL-DY retained DY infectivity (Table 1), and there was no extension in the incubation period compared to untreated SCL-bound control inoculated animals (Table 1). These data are in contrast with the lack of PMCA conversion observed in PMCAsi experiments involving treated SCL. There are several possible explanations for this observation. In bioassay experiments, changes in incubation period are correlated to changes in the titer of inoculum (59–61); however, 10-fold to 100-fold differences in titer can result in similar incubation periods (59, 62, 63). PMCA is more precise than bioassay at measuring changes in infectious PrP^Sc^ units; therefore, it is possible that the observed reduction in PMCA is insufficient for measurement by animal bioassay. Alternatively, it is possible that cellular processes that occur in the animal and not in PMCA can disassociate PrP^Sc^ from SCL, resulting in a higher measured titer by animal bioassay than by PMCA (64). Further experiments are necessary to further define these relationships.

Multiple strains of CWD prions exist in nature; however, the conditions that influence the abundances and distributions of strains are unknown (65–69). Here, we show that degradation or exposure to wetting and drying treatment can enhance the emergence of a more stable, highly pathogenic strain *in vitro.* The potential for multiple prion exposure and binding events should be taken into consideration in modeling prion transmission dynamics.

## MATERIALS AND METHODS

### Ethics statement.

All procedures involving animals were approved by the Creighton University Institutional Animal Care and Use Committee and comply with the Guide for the Care and Use of Laboratory Animals.

### Prion sources and tissue preparation.

Brains from clinically affected hamsters infected with either the Hyper (HY) strain or the Drowsy (DY) prion strain causing hamster-adapted transmissible mink encephalopathy (TME) were homogenized in Dulbecco’s phosphate-buffered saline (DPBS) (Mediatech, Herndon, VA) to 10% (wt/vol) using strain-dedicated tissue grinders (Tenbroeck, Vineland, NJ). Uninfected hamster brain was homogenized to 10% (wt/vol) in PMCA conversion buffer (phosphate-buffered saline [pH 7.4] containing 5 mM EDTA, 1% [vol/vol] Triton X-100, and complete protease inhibitor tablet [Roche Diagnostics, Mannheim, Germany]) using a dedicated Tenbroeck tissue grinder. The brain homogenate was centrifuged at 1,500 × *g* for 30 s, and the supernatant was collected and stored at –80°C.

### Prion adsorption to soil.

HY and DY PrP^Sc^ adsorption to gamma-irradiated silty clay loam (SCL) (farm in Iowa [40°55’08.2”N, 91°10’08.1”W] with no previously reported incidence of prion disease; courtesy of Shannon Bartelt-Hunt) was performed as previously described (29, 70). Briefly, gamma-irradiated SCL was mixed with 10% (wt/vol) brain homogenates and DPBS and rotated at 24 rpm for 24 h at room temperature before being subjected to 5 cycles of centrifugation (1,500 × *g* for 30 s) and washing in DPBS prior to collection of the final pellet.

### Dehydration and rehydration of environmental samples.

Repeated cycles of drying and wetting were performed as described previously (35). Briefly, 20 μl of each sample was placed in an uncapped 200-μl tube (Thermo Scientific) and incubated at 40°C for 12 h and rehydrated with 20 μl of ultrafiltered deionized water. A cycle is defined as one drying cycle followed by rewetting. After 10 cycles, samples were stored at –80°C.

### Proteinase K treatment.

HY-infected and DY-infected (5% [wt/vol]) brain homogenates were incubated with 50 μg/ml of proteinase K (PK) (Roche Diagnostics) for 24 h or subjected to mock digestion in DPBS. After incubation, samples were treated with 100 units/ml of Benzonase (Sigma-Aldrich) at 37°C for 1 h. Benzonase and PK were removed by a series of centrifugation steps (10,000 × *g* for 30 min at 10°C) and washing steps in 20% (wt/vol) N-lauryl-sarcosine (NLS) (3 times) and DPBS (2 times) (10,000 × *g* for 30 min at 10°C and 200,000 × *g* for 1 h at 10°C, respectively) before resuspension of the final pellet in 0.1% (wt/vol) NLS.

### Protein misfolding cyclic amplification.

Protein misfolding cyclic amplification (PMCA) was performed as previously described (71). Samples (*n* ≥ 3) in PMCA conversion buffer were placed into polypropylene tubes in a Misonix 3000 sonicator (Misonix, Farmingdale, NY). The average output of the sonicator was 165 W during each sonication cycle. A PMCA round consisted of 144 cycles of a 5-s sonication, followed by an incubation of 9 min 55 s at 37°C. After each round of PMCA, an aliquot of sonicated sample was added to fresh 10% (wt/vol) uninfected brain homogenate in PMCA conversion buffer before the next round of sonication. The ratio of seed to uninfected brain homogenate was 1:20 for the first round of PMCA, 1:10 for the second round, and 1:2 for the remaining rounds. Aliquots (*n* ≥ 3) of uninfected brain homogenate were included in all rounds of PMCA as a negative control.

### Western blotting.

Detection of PrP^Sc^ by Western blotting was performed as previously described (71). Briefly, PMCA reaction samples were digested with PK at a final concentration of 50 μg/ml (Roche Diagnostics Corporation, Indianapolis, IN) at 37°C for 60 min. Digestion was terminated by boiling samples at 100°C for 10 min in sample loading buffer (4% [wt/vol] SDS, 2% [vol/vol] β-mercaptoethanol, 40% [vol/vol] glycerol, 0.004% [wt/vol] bromophenol blue, 0.5 M Tris buffer, pH 6.8). Samples were size fractionated on 4% to 12% bis-Tris-acrylamide (NuPAGE; Invitrogen, Carlsbad, CA) and transferred to a polyvinylidene difluoride (PVDF) membrane (Immobilon P; MilliporeSigma, MS). Membranes were incubated with 5% (wt/vol) nonfat dry milk–Tween Tris-buffered saline (TTBS) (Bio-Rad Laboratories, Hercules, CA) for 30 min. Mouse monoclonal anti-PrP antibody 3F4 (Chemicon, Temecula, CA) (0.1 μg/ml) was used to detect hamster prion protein. Western blots were developed with Pierce SuperSignal West Femto maximum-sensitivity substrate (Pierce, Rockford, IL) and imaged using a Li-Cor Odyssey Fc imaging system (Li-Cor, Lincoln, NE) or Kodak 4000R imaging station (Kodak, Rochester, NY) as previously described (71). PrP abundance was quantified using Kodak molecular imaging software v.5.0.1.27 (Kodak, New Haven, CT) or Li-Cor Image Studio software v.1.0.36 (Li-Cor, Lincoln, NE). Migration analysis of the unglycosylated PrP^Sc^ polypeptide was determined using NIH ImageJ Fiji software (NIH, USA) and the lane analysis function.

### Statistical analysis.

Two-tailed Student’s *t* tests and one-way analysis of variance (ANOVA) were carried out using Prism 7 software (GraphPad Software Inc., San Diego, CA). A value was considered statistically significant if the *P* value was less than or equal to 0.05. One-way ANOVA and Tukey’s multiple-comparison test were used for analyzing animal bioassay data.
