# Supplementary figures and images for: Alteration of Prion Strain Emergence by Nonhost Factors
Source: mSphere. 2019 Oct 9;4(5):e00630-19. doi: 10.1128/mSphere.00630-19 (PMC6796975; doi:10.1128/mSphere.00630-19)

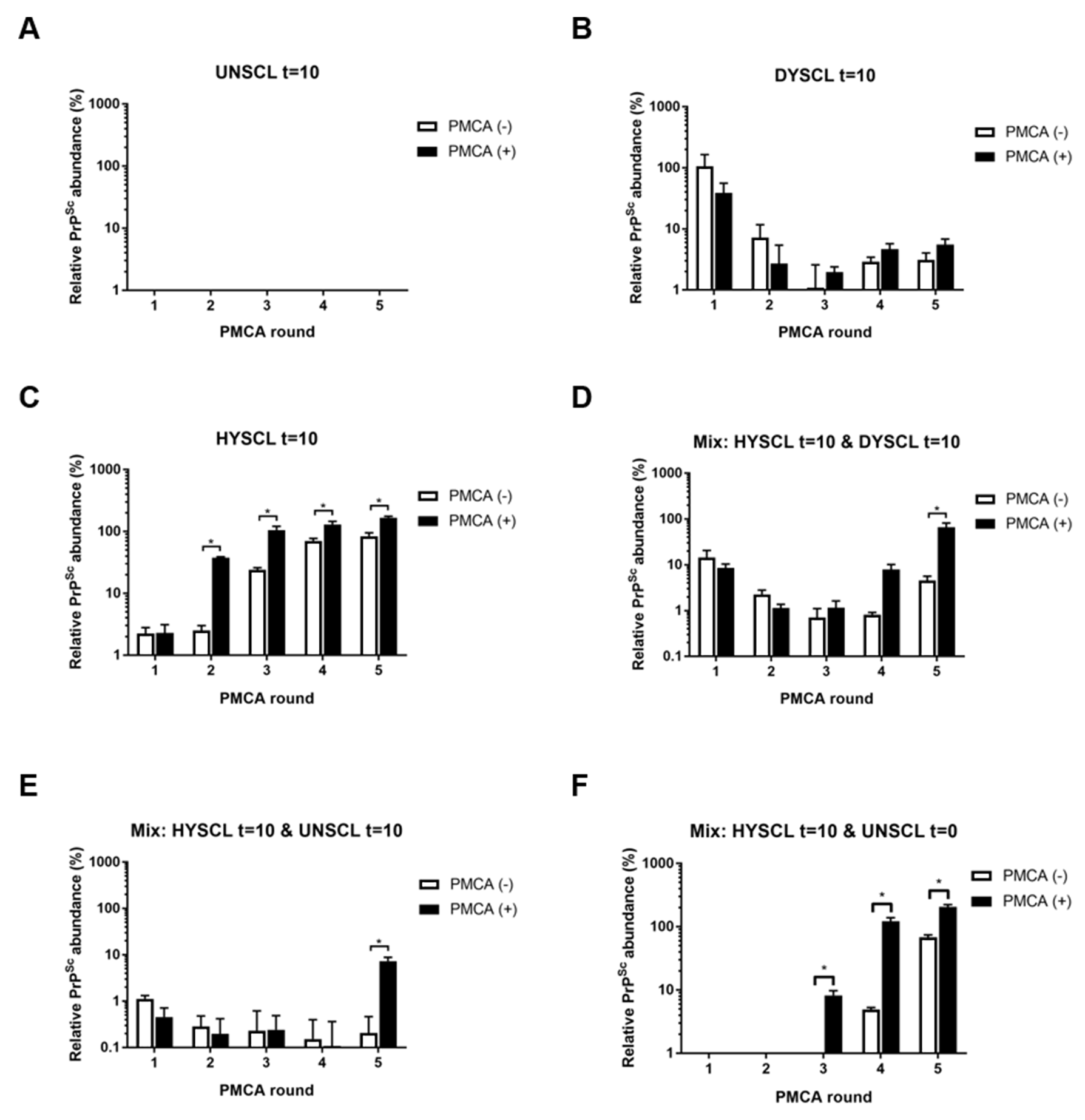

Supplement: FIG S1 [file mSphere.00630-19-sf001.tif]

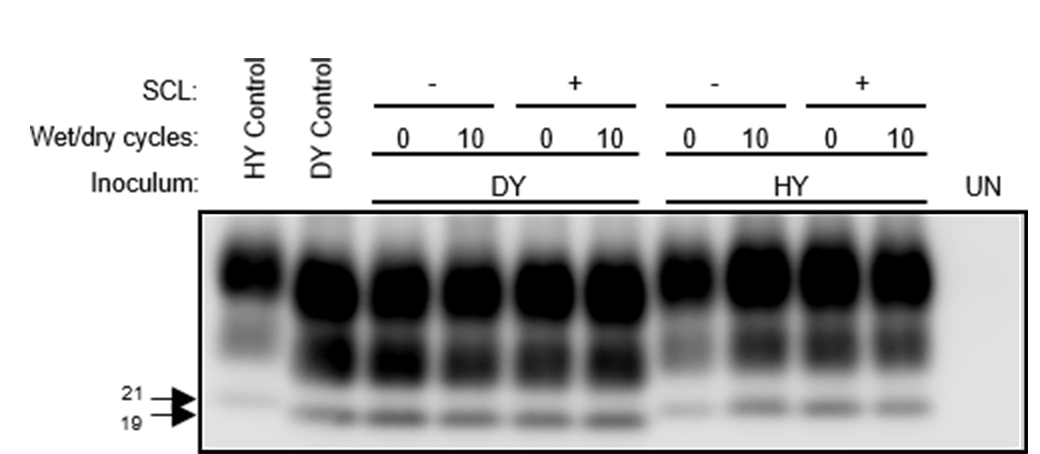

Supplement: FIG S2 [file mSphere.00630-19-sf002.tif]
